# Mechanical characteristics of three staples commonly used in foot surgery

**DOI:** 10.1186/1757-1146-2-5

**Published:** 2009-02-25

**Authors:** Ulfin Rethnam, Jan Kuiper, Nilesh Makwana

**Affiliations:** 1 Department of Orthopaedics, Glan Clwyd Hospital, Bodelwyddan, UK; 2 Hip Research Unit, Robert Jones & Agnes Hunt Orthopaedic Hospital, Oswestry, UK; 3 Department of Orthopaedics, Robert Jones & Agnes Hunt Orthopaedic Hospital, Oswestry, UK

## Abstract

**Background:**

Bone staples are an accepted method of fixation in foot surgery. They reduce operating time and trauma in surgical procedures. A variety of memory staples are available but their properties compared to standard staples are not known. We carried out a study comparing two popular types of memory staples and a standard stainless steel staple.

**Methods:**

Standardized bone models of metatarsals made from Tufnol tubes were osteotomized and stabilised using one of three types of bone staples, two types of memory staple (Memory staple and heat-activated Memoclip) or a standard stainless steel staple (Richards). Constructs were loaded in bending and torsion on a material testing machine. The moment and torque to achieve 10 degree of bending or torsion and permanent angulation of the osteotomized bones were assessed.

**Results:**

The Richards staple was found to provide a four times larger resistance to bending and torsion than the two memory staples. However, it was permanently deformed after bending. The Memory and Memoclip staples were equal in their stiffness. In addition, angulation of bones fixed with the Memoclip was elastic, preventing any permanent deformation.

**Conclusion:**

The Richards staple was stiffer, although the permanent deformation of this staple is a disadvantage. Memoclip staples exhibit lower but adequate stiffnesss when compared to the standard Richards staple and are not permanently deformed after bending. The Memoclip staples were easier to handle. The results will enable surgeons to determine the optimal staple for foot and ankle procedures.

## Background

Bone staples are widely used in foot and ankle surgery. They are considered to be an effective internal fixation method [1]. The cited advantages of bone staples include easier fixation onto bones, reduced surgical time and trauma. This in turn leads to improved healing and reduced post-operative pain [2].

Various types of bone staples are available, differing in their shape and physical properties. Recently, bone staples have been introduced based on shape-memory materials. For all staples, manufacturers cite advantages in terms of ease of use and provide compression strength based on physical properties of the metals used to produce the staples. However, little is known about the actual fixation capabilities of staples used in foot and ankle surgery and how these compare between staples. It is unknown how the fixation capabilities of staples based on memory material compare to those of conventional metals.

The aim of this study was to compare two types of staples based on memory metal and one conventional staple, all three commonly used in foot and ankle surgery. Specifically, we addressed the question regarding the bending stiffness, torsional stiffness, and permanent deformation of these staples. The null hypothesis was that there was no difference in stiffness between the staples.

## Methods

This study was conducted in the Laboratory for Biomechanics at The Robert Jones and Agnes Hunt Orthopaedic Hospital in Oswestry after approval from the review board.

### Materials

**Figure 1:**
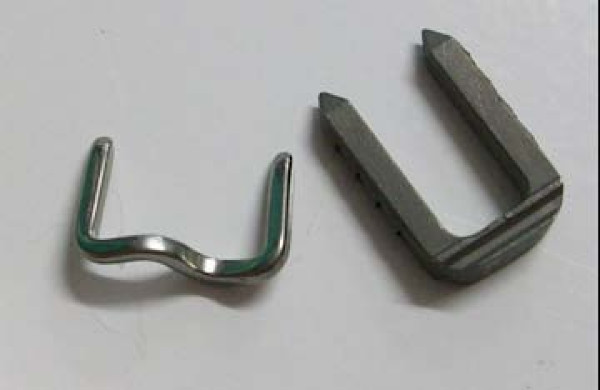
**Memory (left) & mechanical (right) staples**.

Three types of staples were used in this study, two of which were made of nickel-titanium (nitinol) memory material. All the staples had a bridge dimension of 14 mm and leg dimensions of 12 mm. The first of these, Memoclip (Heritage, United Kingdom), is heat activated (Figure 1). When the staple is exposed to a temperature above 50 degrees Centigrade, these staples deform their limbs and generate compression. The second of these, the Memory staple (Depuy, United Kingdom), is normally stored at 0 – 4 degrees Centigrade. When the staple is brought to body temperature (37 degrees Centigrade), the limbs deform and cause compression of bone. The third staple (Richards staple, Smith & Nephew, United Kingdom) is made of stainless steel (Figure 1). Compression is achieved by the mechanical properties of the staple. No further compression is achieved once the staple is inserted into bone.

### Specimen preparation

**Figure 2:**
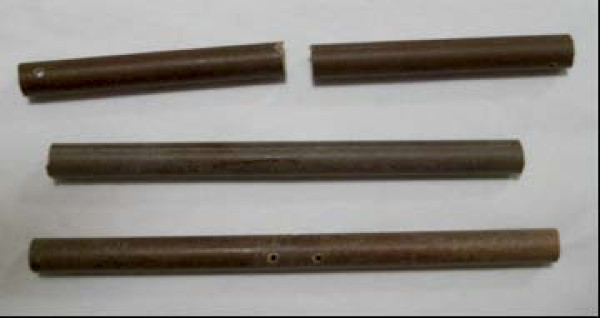
**Tufnol tubes used**.

Standardised metatarsal bone models made of Tufnol tubes (10 mm diameter) were used (Figure 2). We used Tufnol tubes for our study as they exhibit similar properties to bone and are commonly used in biomechanical studies [3,4].

The Tufnol tubes were 15 cm in length and they were osteotomised in the centre with a saw. The osteotomised tubes were stabilised using a single staple applied perpendicular to the osteotomy. Fixation of the near and far cortices (bicortical) was done for each specimen. 5 specimens were constructed for each staple type (n = 5 for each staple type).

### Mechanical testing

**Figure 3:**
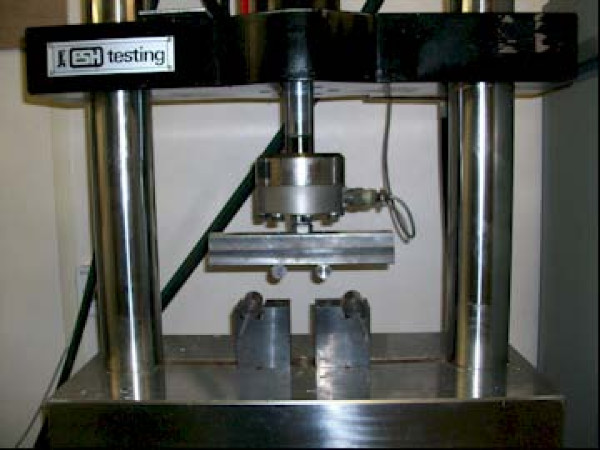
**Materials testing machine**.

**Figure 4:**
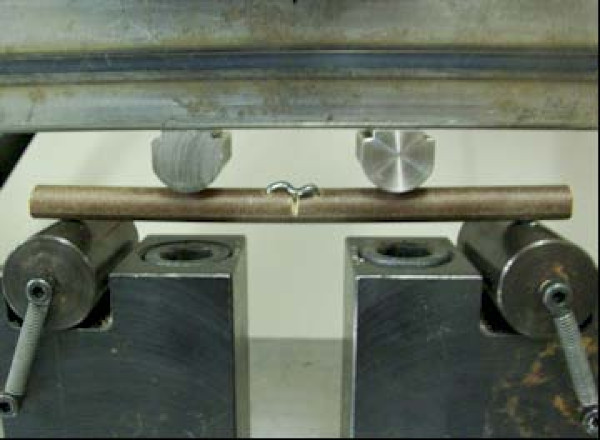
**Specimen undergoing four point bending stress**.

The prepared specimens were mounted on a materials testing machine (ESH Ltd, Brierly Hills, United Kingdom) (Figure 3). Each construct was tested in four point bending and torsional stress (Figure 4). Load displacement curve and stiffness of fixation of each staple were measured. A constant temperature of 37 degrees Centigrade was maintained throughout the experiment using a thermostat controlled fan heater.

### Outcome measures and statistical analysis

**Figure 5:**
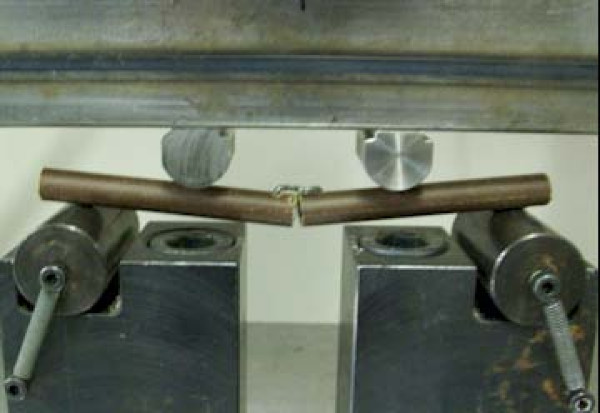
**Deformation of construct on four point bending stress**.

The primary outcome measure was the moment to achieve 10 degree of bending or torsion at the osteotomy site of the specimen. A 10 degree deformation of the construct was considered as mechanical failure of the staple as in a clinical situation 10 degree angulation at a fracture or osteotomy after fixation would be considered as an implant failure (Figure 5).

One-way Analysis of Variance (ANOVA) was used for statistical analysis of the results and the Tukey test was used for pairwise comparison between the staples. p < 0.05 was considered to be statistically significant.

## Results

### Bending stress

**Figure 6:**
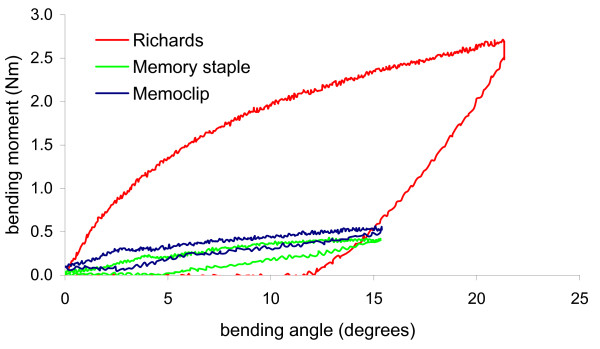
**Load displacement curves for different staples in bending**.

**Figure 7:**
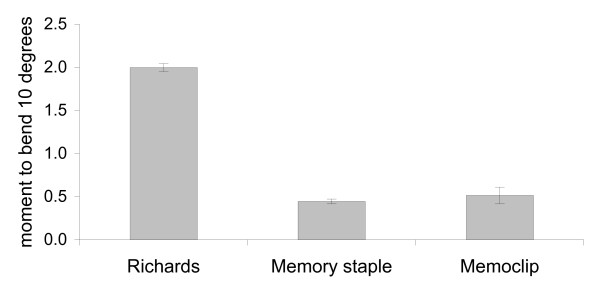
**Bending stiffness for different staples**.

In four point bending, there was a non-linear relation between the bending angle and the bending moment. The average moment to achieve 10 degree of bend at the osteotomy site of the specimens was as follows: Richards staple: 1.9 ± 0.21 Nm, Memoclip staple: 0.51 ± 0.1 Nm and Memory staple: 0.44 ± 0.03 Nm. The Richards staple was four times stiffer than the Memory and Memoclip staples. This difference was statistically significant (p < 0.01, ANOVA followed by Tukey pairwise test) (Figure 6, 7). There was no significant difference between the Memoclip and Memory staple (p = 0.26, ANOVA followed by Tukey pairwise test). On removal of the load, constructs fixed with the Memoclip staple returned to their original shape while the other two staples were permanently deformed (Figure 6).

### Torsional stress

In torsion, there was a linear relation between the torsion angle and applied torque. The average moment to achieve 10 degree torsion of the construct was as follows: Richards staple: 0.12 ± 0.03 Nm/degree, Memoclip staple: 0.04 ± 0.01 Nm/degree and Memory staple: 0.03 ± 0.01 Nm/degree.

**Figure 8:**
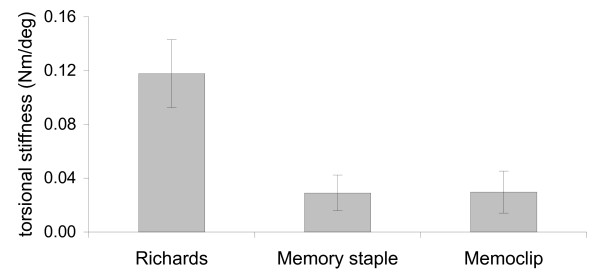
**Torsion stiffness for different staples**.

The Richards staple was four times stiffer than the Memory and Memoclip staples. This difference was statistically significant (p < 0.01, ANOVA followed by Tukey pairwise test) (Figure 8). There was no significant difference in torsional stiffness between the Memoclip and the Memory staple (p = 0.998, ANOVA followed by Tukey pairwise test). Upon removal of the load, all constructs returned to their original shape suggesting that there was no permanent deformation of the staples.

## Discussion

Bone staples are used commonly in foot and ankle surgery. No data exists in the literature on the comparative analysis of biomechanical properties of bone staples. The fixation stiffness in bone staples is one of the most important factors determining the success of osteosynthesis along with compression achieved at the osteotomy site. There are studies evaluating the mechanical properties using different staple configuration and leg lengths of staples [5]. There are no studies that compare mechanical properties of staples with different physical properties.

The staples selected for the study were made of different materials and had different physical properties. Two staples (Memoclip and Depuy Memory staple) achieved compression following deformation of the limbs when exposed to a particular temperature. The other staple (Richards) achieved compression by mechanically holding the bone ends across the osteotomy site. With bicortical fixation, the constructs fixed with the Richards staple was the stiffest in both bending and torsion stress. There was a permanent deformation of the Richard staple on bending and this may be a disadvantage of this staple as deformation of this manner in a clinical scenario would cause distraction at the osteotomy site.

Both temperature activated memory staples (Memoclip and Memory staple) had similar fixation stiffness in bending & torsion. The Memoclip staple resisted permanent deformation while the Memory staple revealed some permanent deformation on bending. The advantages of these staples are that they cause compression at the osteotomy site after insertion. The mechanically activated Richards staple will also apply compression after insertion; it will not however have a propensity to deform further as will the shape-memory staples. Comparing the two memory staples, the Memoclip staples were easy to use as deformation of the limbs occurred only when exposed to temperatures >50 degree Centigrade. The Memory staple showed deformation of the limbs when exposed to body temperature therefore making insertion more difficult.

The strength of our study was that it was conducted in a uniform environment maintaining constant temperature and a substitute (Tufnol tubes) similar to bone was used.

The results of our study show that permanent deformation of the Richards staple occurs with increasing bending and torsional stress. However the Richards staple was on an average four fold stiffer than the temperature dependent staples. There was no significant difference between the Memoclip and Memory staples with regards to stiffness although the Memory staple did undergo permanent deformation in bending. This study will allow surgeons to make a more informed choice when using staples in foot and ankle surgery. Further clinical trials would be needed to compare these staples with regards to bone healing and fatigue failure.

## Conclusion

Biomechanical testing of three different staples used in foot and ankle surgery revealed the Richards staple to have the highest fixation stiffness in both bending and torsion, although there was permanent deformation. The Memoclip staples provided adequate stiffness, resisted permanent deformation and were easier to handle which could be its advantages when compared to the Richards and Memory staple.

## Competing interests

The authors declare that they have no competing interests.

## Authors' contributions

UR the main author for the manuscript and was involved in specimen preparation, conducting the study, data acquisition and preparation of the manuscript. JK was involved in setting up of equipment, data analysis, interpretation of data and preparation of the manuscript. NM the senior author was involved in conception and design of the study. All authors have read and approved the final manuscript.

## About the Authors

UR - Orthopaedic registrar. JK - Research scientist. NM - Consultant Trauma & Orthopaedics
